# Role of Prolactin Receptors in Lymphangioleiomyomatosis

**DOI:** 10.1371/journal.pone.0146653

**Published:** 2016-01-14

**Authors:** Amira Alkharusi, Elena Lesma, Silvia Ancona, Eloisa Chiaramonte, Thomas Nyström, Alfredo Gorio, Gunnar Norstedt

**Affiliations:** 1 Department of Clinical Science and Education, Södersjukhuset, Karolinska Institutet, Stockholm, Sweden; 2 Center for Molecular Medicine, Karolinska Institutet, Stockholm, Sweden; 3 Department of Women’s and Children’s Health, Karolinska Institutet, Stockholm, Sweden; 4 Department of Health Sciences, Laboratories of Pharmacology, Università degli Studi di Milano, Milano, Italy; 5 Sultan Qaboos University, College of Medicine and Health Sciences, Muscat, Oman; Duke University Medical Center, UNITED STATES

## Abstract

Pulmonary lymphangioleiomyomatosis (LAM) is a rare lung disease caused by mutations in the tumor suppressor genes encoding Tuberous Sclerosis Complex (TSC) 1 and TSC2. The protein product of the TSC2 gene is a well-known suppressor of the mTOR pathway. Emerging evidence suggests that the pituitary hormone prolactin (Prl) has both endocrine and paracrine modes of action. Here, we have investigated components of the Prl system in models for LAM. In a TSC2 (+/-) mouse sarcoma cell line, down-regulation of TSC2 using siRNA resulted in increased levels of the Prl receptor. In human LAM cells, the Prl receptor is detectable by immunohistochemistry, and the expression of Prl in these cells stimulates STAT3 and Erk phosphorylation, as well as proliferation. A high affinity Prl receptor antagonist consisting of Prl with four amino acid substitutions reduced phosphorylation of STAT3 and Erk. Antagonist treatment further reduced the proliferative and invasive properties of LAM cells. In histological sections from LAM patients, Prl receptor immuno reactivity was observed. We conclude that the Prl receptor is expressed in LAM, and that loss of TSC2 increases Prl receptor levels. It is proposed that Prl exerts growth-stimulatory effects on LAM cells, and that antagonizing the Prl receptor can block such effects.

## Introduction

Lymphangioleiomyomatosis (LAM) is a rare, progressive multisystem disease affecting women of child bearing age which is characterized by proliferation of abnormal smooth muscle (SM)-like LAM cells [1]. LAM patients are usually fertile females and a common disease manifestation is the appearance of SM tumours, i.e. LAM lesions, in the lung parenchyma [2]. Loss of function of the tuberous sclerosis complex 2 (TSC2) gene has been observed in LAM [3]. Recent research has shown that the TSC2 gene encodes a protein that suppresses the mTOR pathway [4–8]. The mTOR pathway affects several key cellular functions including protein synthesis. Endocrine components of LAM have been suspected because females are predominantly affected, and because the disease is aggravated during pregnancy–a condition associated with elevation of lactogenic hormones like prolactin [9,10].

Prl exerts a variety of functions related to growth, differentiation and metabolism in different target tissues [11]. The hormone is most well-known as a lactogenic factor secreted from the pituitary gland, but it is also clear that some functions of Prl are mediated by localized, non-pituitary Prl production [12]. Extra-pituitary actions of Prl seem particularly relevant in humans because of the presence of a separate promoter within the Prl gene [12,13]. Prl signals via the Prl receptor (PrlR), a member of the cytokine receptor family. The PrlR is structurally related to the GH receptor (GHR), and hormone binding leads to receptor dimerization and activation of the JAK2-STAT-SOCS pathway [14]. Prl seems to only bind to the PrlR, whereas GH can bind to both GHR and the PrlR.

GH and Prl stimulate cell proliferation and growth. Besides activating the STAT system, these hormones stimulate protein synthesis, an effect for which the kinase AKT-mTOR-S6 plays an important role [15,16]. One prerequisite for cells to respond to GH/Prl is the level of expression of the GH/Prl receptors, and suppressor of cytokine signalling 2 (SOCS2) is an important regulator of GHR expression levels [17]. Knock-down of SOCS2 in cells and animals increases the levels of GHR expression, leading to increased GH sensitivity [18,19].

Since LAM is related to increased activation of the mTOR system, attention has been focussed on treatment with rapamycin analogues, and promising effects on the disease progression have been reported [20–22]. In this study, we have analysed the relationship between TSC2 and the Prl system both in cell systems in which TSC2 expression levels have been altered by siRNA transfection, and also in cell lines derived from LAM patients.

## Materials and Methods

### Cell culture, cell lysis and protein quantification

The cell line CRL-2620, obtained from American Type Culture Collection (ATCC) and originating from a TSC2 -/+ mouse sarcoma, was cultivated in Dulbecco's Modified Eagle's Medium (DMEM) supplemented with 10% fetal bovine serum (FBS) (Gibco), 100 U/ml penicillin and 100 μg/ml streptomycin, at 37°C in an atmosphere of 5% CO_2_.

Human cells were isolated from patients with LAM/TSC who had given written informed consent according to the Declaration of Helsinki. The study was approved by the Institutional Review Board of Milan’s San Paolo Hospital. LAM/TSC cells were previously characterized as a homogenous population of α-smooth muscle-like (ASM) cells. These cells have a mutation in one TSC2 allele, and loss of function of the other TSC2 allele is suggested by the absence of any TSC2 product [23]. The cells were grown in a 50:50 mixture of DMEM/Ham’s F12 (Gibco) supplemented with hydrocortisone (2.5 ug/ml), EGF (10 ng/ml), sodium selenite (8.6 ng/ml), insulin (25 ug/ml), transferrin 10 ug/ml), ferrous sulphate (0.445 ug/ml) and 15% FBS. Cells were used between passages 5 and 15.

Cells were lysed in 50 mM Tris HCl, pH 7.5/150 mM NaCl/5 mM EDTA/0.5% Igepal NP-40/1 mM Na_3_VO_4_/20 mM NaF/1 mM DTT/1 mM PMSF/1× protease inhibitor cocktail (Complete mini, Roche). Cell debris was removed by centrifugation at 14,000× g for 15 minutes at 4°C. The concentration of Prl hormone for treatment was 200ng/ml, unless otherwise specified. The protein content of the supernatant was determined using the bradford dye-binding method [24].

### Cell transfection, siRNA treatment of cells and quantitative PCR

Cell transfections using siRNAs directed against TSC2 and control siRNA were purchased from QIAGEN. Electroporation, using AMAXA nucleofector technology (Lonza, Cologne, Germany) was carried out using TSC2 siRNA with the sequence TTGCTGGAAGTTGATGCGTAA, and a scrambled siRNA sequence was used as a control. Transfections were carried out using 20nmol siRNA on 1.5 million cells with an Amaxa instrument (setting E14). Quantitative RT-PCR was used to measure changes in TSC2 mRNA levels.

### Western blot

Whole cell lysates were separated in SDS/PAGE gels and transferred to polyvinylidenediflouride (PVDF) membranes (Millipore). After blotting, membranes were blocked in 5% non-fat skimmed milk or BSA (Sigma–Aldrich, St. Louis, Missouri, USA) in Tris-Buffered Saline (TBS) containing 0.1% Tween 20. Membranes were incubated with one or more of the following antibodies, as specified in the figures: mouse anti-GHR B10 (Santa Cruz Biotechnology, Santa Cruz, CA, USA), rabbit anti-PrlR M-170 (Santa Cruz Biotechnology, Santa Cruz, CA, USA). Human samples were probed with PrlR antibody clone 1A2B1 (Invitrogen, Carlsbad, CA, USA). Antibodies to detect phosphorylated and un-phosphorylated STAT5, STAT3 and Erk were obtained from Cell Signaling (Danvers, Massachusetts, USA). As a loading control, GAPDH horseradish peroxidase conjugated antibody (Cell Signaling, Danvers, Massachusetts, USA) was used. Primary specific HRP-conjugated secondary antibodies were obtained from Santa Cruz Biotechnology (Santa Cruz, CA, USA). Membranes were visualized with an ECL Western blotting detection system (Pierce, ThermoFisher Scientific Inc, Rockford, IL, USA) according to the manufacturer's instruction, or using an Amersham ECL Prime Western Blotting Detection Reagent from GE healthcare. Band intensities were assessed by densitometry, and phospho-protein levels were normalized to the total levels of each respective protein.

### Immunofluorescence staining and confocal laser scanning

For immunofluorescence staining, cells were grown on coverslips and cultured in the supplemented media at 37°C in an atmosphere of 5% CO_2_. Cells were fixed with ice-cold methanol for 10 min, after which excess methanol was removed. After three subsequent washes with PBS, cells were blocked and permeabilized for 1 hour with PBS containing 1% BSA, 5% goat serum and 0.05% Triton X-100 (Sigma–Aldrich, St. Louis, Missouri, USA). The cell coverslips were incubated with primary antibody (mouse anti-GHR B10 (Santa Cruz, CA, USA), mouse anti-PrlR, clone 1A2B1 (Invitrogen, Carlsbad, CA, USA) and rabbit anti-PrlR M-170 (Santa Cruz, CA, USA) overnight at 4°C, washed again 3 times with PBS, and incubated for 1 h at room temperature in the dark with the secondary antibodies alexa fluor 488 goat anti-mouse and alexa flour 594 goat anti-rabbit (both from Invitrogen, Carlsbad, CA, USA). Finally, the slides were washed, mounted with Vectashield containing DAPI (H-1200, Vector Laboratories, CA, USA) and stored at 4°C in the dark. Controls for specificity and background staining were performed by incubating with mouse IgG or rabbit IgG antibodies. Image acquisition was performed by confocal laser scanning microscopy (CLSM).

### Immunohistochemistry analyses

Lung tissue samples were taken from patients with a diagnosis of LAM as confirmed by pulmonary histopathology using both hematoxylin-eosin (H&E) staining, and also specific studies (e.g., reactivity with monoclonal antibody HMB45). Tissue was obtained at lung transplantation or following lung biopsy for medically indicated reasons. Tissues from patients were used after informed consent according to the Declaration of Helsinki.

Immunohistochemistry was carried out on de-paraffinized rehydrated 4 μm tissue sections. For antigen retrieval, the slides were treated under pressure at 95°C in citrate buffer, pH 6.0 (Dako, Glostrup, Denmark) for 5 minutes. After cooling to room temperature, the sections were incubated with 0.3% H_2_O_2_ in methanol for 20 minutes to quench endogenous peroxidase activity, and then incubated in TBS with 3% bovine serum at room temperature for 2 hours to block non-specific binding. Sections were incubated at 4°C overnight with a primary antibody against PrlR (1:100; Invitrogen, Carlsbad, CA, U.S.A.) diluted in 1.5% BSA. The sections were then incubated with biotinylated secondary antibody (1:100; Vector Laboratories) for 2 hours at room temperature, followed by incubation with Avidin-Biotin-Peroxidase Complex (Pierce, Rockford, IL, USA). Detection was performed with peroxidase substrate DAB (3.3-diaminobenzidine) (DAB substrate kit, Pierce). Haematoxylin was used as a counterstain. For negative controls, the primary antibody was omitted. Negative controls were run in parallel in all experiments. Images were captured by bright field microscopy under identical conditions of magnification and illumination.

### Cell proliferation assay

Cells were seeded in 96-well culture plates at a density of 1000 cells per 200μL culture medium per well, and allowed to attach for 24–48 hours. The media was then removed and replaced with serum-free medium, or medium containing 2% or 10% serum and 200ng/ml recombinant human Prl (a generous gift from Novo Nordisk A/S, Denmark), with or without PrlR antagonist (PrlRA) as specified in the Figure legend. Relative cell numbers were assessed after 72 hours, using the colorimetric crystal violet assay. The cells were fixed within the wells by addition of 100μL 4% paraformaldehyde solution to 100μL medium. After 20 minutes, the formaldehyde was discarded, and the plates were washed with deionized water, air-dried, and stained with 100μl crystal violet solution (0.04% (w/v) crystal violet in 1% ethanol) for 20 minutes at room temperature. Excess dye was washed away with deionized water and the plates were air-dried. Bound dye was then dissolved with 1% (w/v) SDS, and the optical densities of the extracts were measured at 600 nm. Growth curves were created on the basis of the obtained absorbance values.

### Invasion assay

Human cells, isolated from a patient with LAM/TSC, were starved overnight. The invasive properties of cells were analyzed using the CytoSelect^™^ Cell Invasion Assay kit (Cell Biolabs, San Diego, CA), according to the manufacturer’s instructions. The assay kit includes polycarbonate membrane inserts, which serve as a barrier to discriminate invasive from non-invasive cells. This analysis measures the invasive properties of cells, and depends on the degradation of matrix proteins covering a polycarbonate membrane and cellular passage through the membrane pores. The invading cells at the bottom of the polycarbonate membrane were stained with a cell stain solution (supplied with the kit), and the cells were extracted using an extraction solution. The optical densities of the extracts were measured at 560 nm. Growth charts were created on the basis of the obtained absorbance values.

### Prl receptor antagonist (PrlRA)

As previously shown, an amino acid substitution at position 129 in human Prl of a Gly for an Arg generates a protein (hPrl-G129R) that blocks the Prl receptor [24,25]. Studies have shown that Prl G129R competes with exogenous Prl when added in >10-fold molar excess to compensate for its lower affinity for the PrlR [26,27]. In the present study, we used PrlRA, a protein with increased receptor affinity due to the substitutions S33A, Q73L, G129R and K190R, to block the receptor [28]. This protein was expressed and purified from *E*.*coli* cultures.

### Statistical analysis

Routinely, treatments were performed in triplicates, and experiments were repeated at least three times on several occasions. Statistical significance of the differences was evaluated using unpaired, 2-tailed Student’s*t*-test, and was considered significant when the significance level of the test was *p* < 0.05.

## Results

### Increased expression of PrlR after treatment of CRL-2620 mouse cell line with TSC2 siRNA

**Fig 1:**
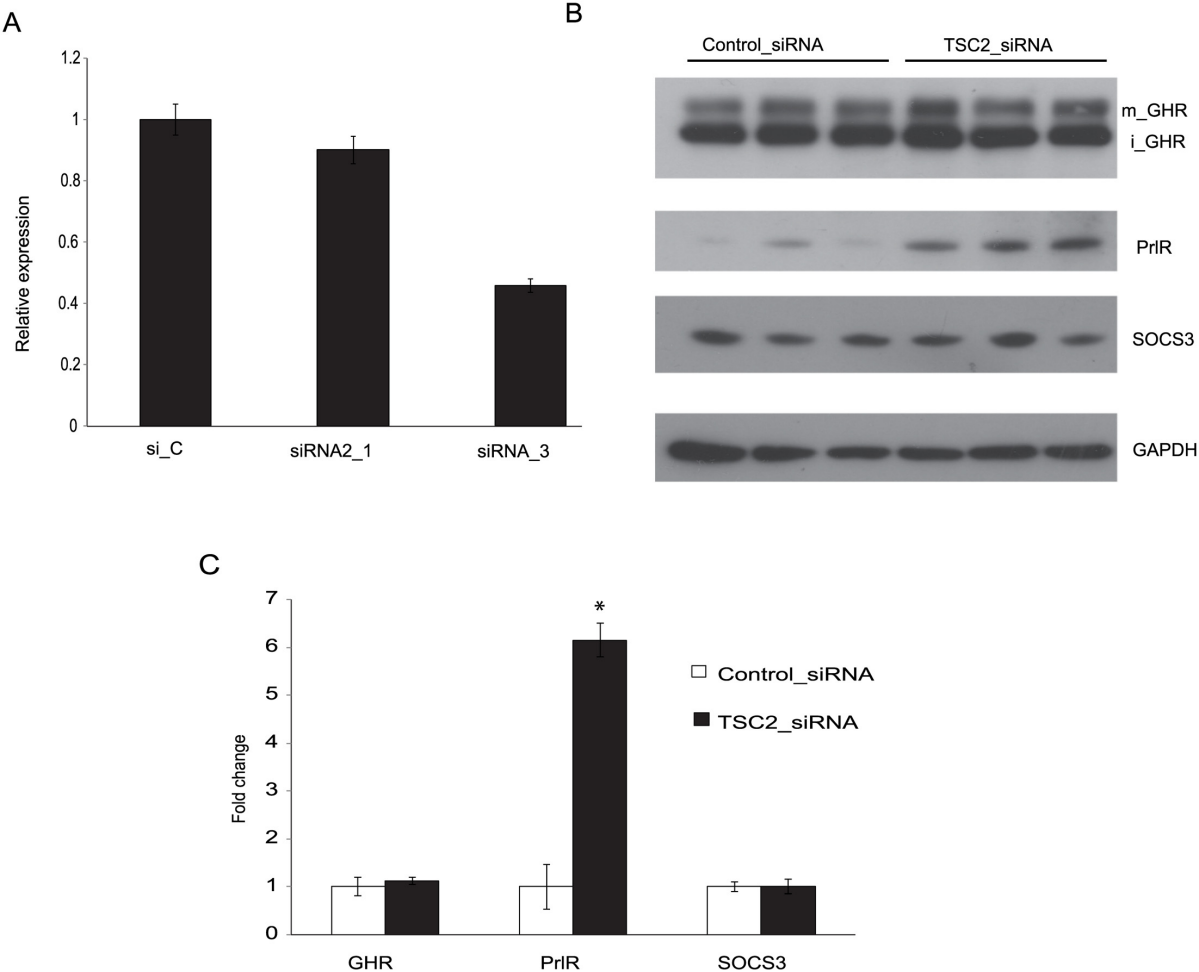
Effect of TSC2 siRNA on GHR and PrlR expression. The mouse cell line CRL-2620 was transfected with two different TSC2 siRNAs (siRNA_1 and siRNA_3), or a control siRNA (si_C). (A) Quantitative RT-PCR was used to test the efficacy with which these siRNAs down-regulated TSC2 mRNA. The most pronounced effect (approximately 60% reduction) was seen with TSC2 siRNA_3, which was used for further experiments. (B) The figure shows a representative Western blot probed with antibodies directed to GHR, PrlR, SOCS3 and GAPDH; iGHR and mGHR, are the immature and mature forms of GHR, respectively. (C) Densitometric analyses of Western blots. Protein lysates were prepared individually from three separate culture dishes treated with TSC2 siRNA or control siRNA and subject to Western blot analysis; experiments were repeated three times and gave consistent results. * = P-value <0.05.

CRL-2620 was originally obtained from a cutaneous sarcoma taken from a TSC2 +/- mouse. Since this cell line is TSC2 (+/-), siRNA was used to knock down remaining TSC2 expression. Quantitative RT-PCR was used to test the efficacy of two different TSC2 siRNAs. Both siRNAs down-regulated TSC2 mRNA levels, with the most pronounced effect (approximately 60% down-regulation) being seen with TSC2 siRNA_3. This was used in further experiments (Fig 1A).

The Western blot technique was used to monitor protein expression following TSC2 siRNA treatments. The levels of PrlR expression were increased in cells treated with TSC2 siRNA (Fig 1B). On the other hand, TSC2 siRNA treatment did not change the levels of GHR. The changes in protein levels were quantified by densitometry, and TSC2 siRNA treatment increased Prl receptor expression by a factor of six-fold (Fig 1C).

**Fig 2:**
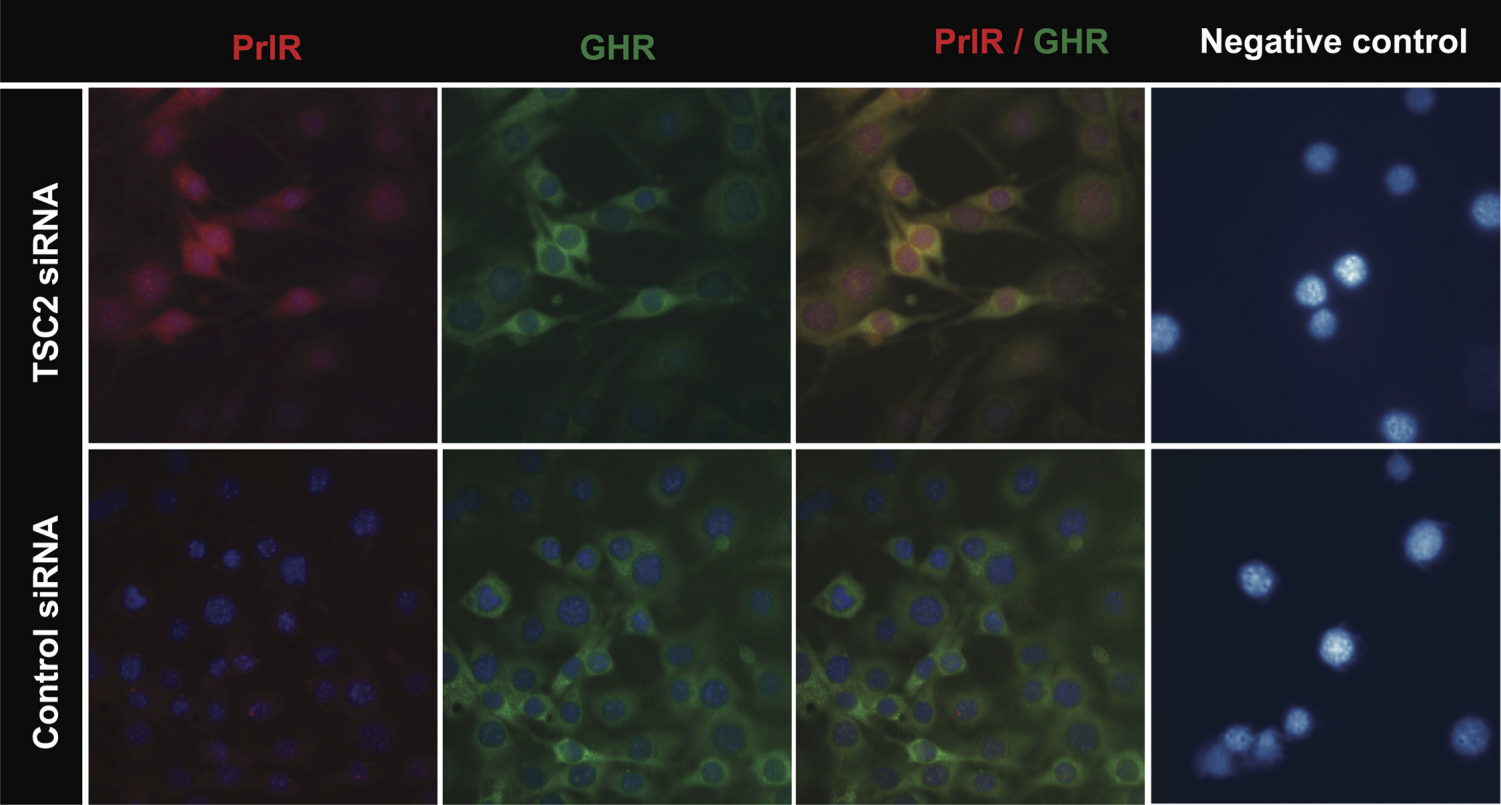
Immunofluorescence of CRL-2620 using PrlR and GHR antibodies. Cells were transfected with TSC2 or control siRNAs. After over-night incubation, the cells were washed 3 times with PBS and incubated with antibodies for PrlR and GHR. The figures show that PrlR immunostaining was markedly increased following TSC2 knock-down. A strong immunofluorescence signal was observed at intracellular locations. In the negative control panel, cells were incubated with mouse IgG and rabbit IgG antibodies. Fluorescence images were acquired at a magnification of 63x.

The above findings were confirmed by immunohistochemical staining studies of cells treated with TSC2 siRNA, compared with control siRNA. Images were acquired by confocal laser microscopy, as shown in (Fig 2). PrlR immunostaining was markedly increased following TSC2 knockdown. A strong immunofluorescence signal was observed at intracellular locations, including the nucleus. GHR immune reactive\ity was also assessed in the same specimen, and is shown in (Fig 2). These experiments confirmed that PrlR staining was increased in the presence of reduced TSC2 levels, while GHR staining was not altered.

### Detection of PrlR in human LAM/TSC cells and in human LAM tissue

**Fig 3:**
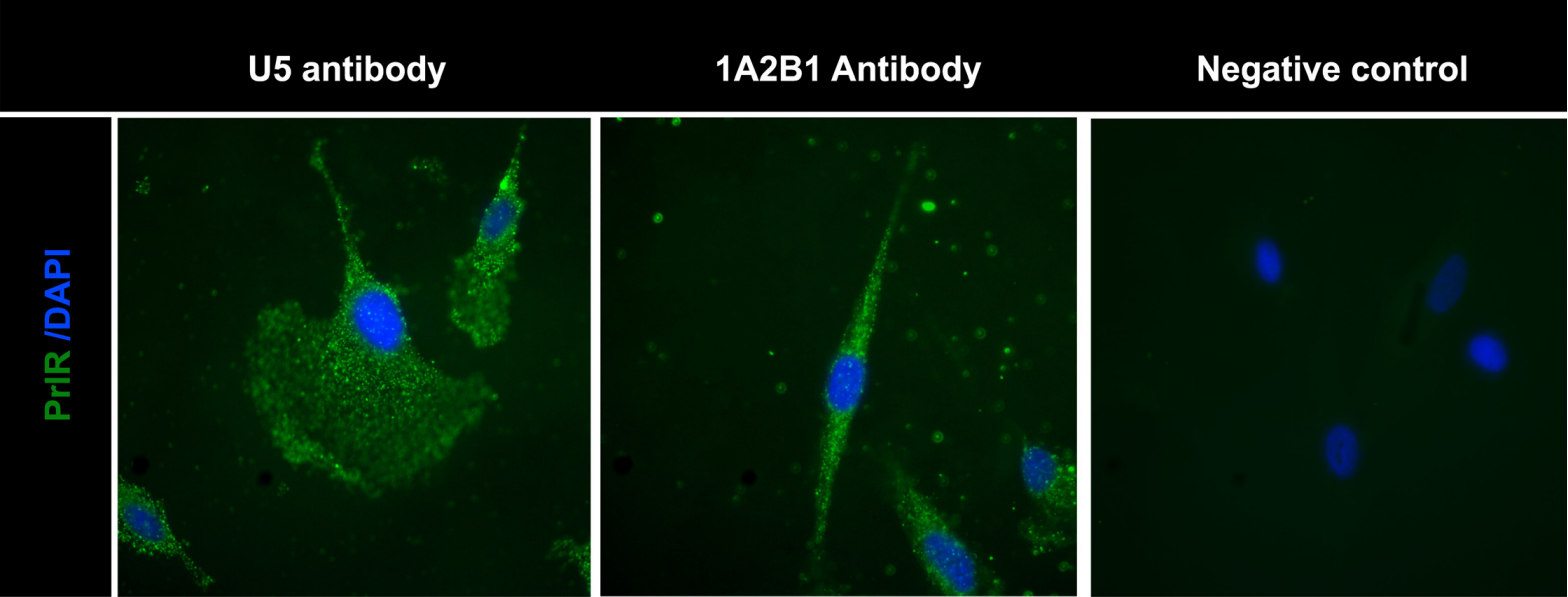
Detection of PrlR in human LAM cells using immunofluorescence. Staining LAM/TSC cells using different PrlR antibodies detect a significant immunofluorescence signal in LAM/TSC cells. The antibodies employed were U5 (Thermoscientific) and A12B1 (Invitrogen).

Human LAM/TSC cells were used for further experiments. Analyses of PrlR expression in these cells were carried out by immunohistochemistry using two different anti-PrlR antibodies, and, as shown in (Fig 3), both antibodies gave a clear staining pattern.

**Fig 4:**
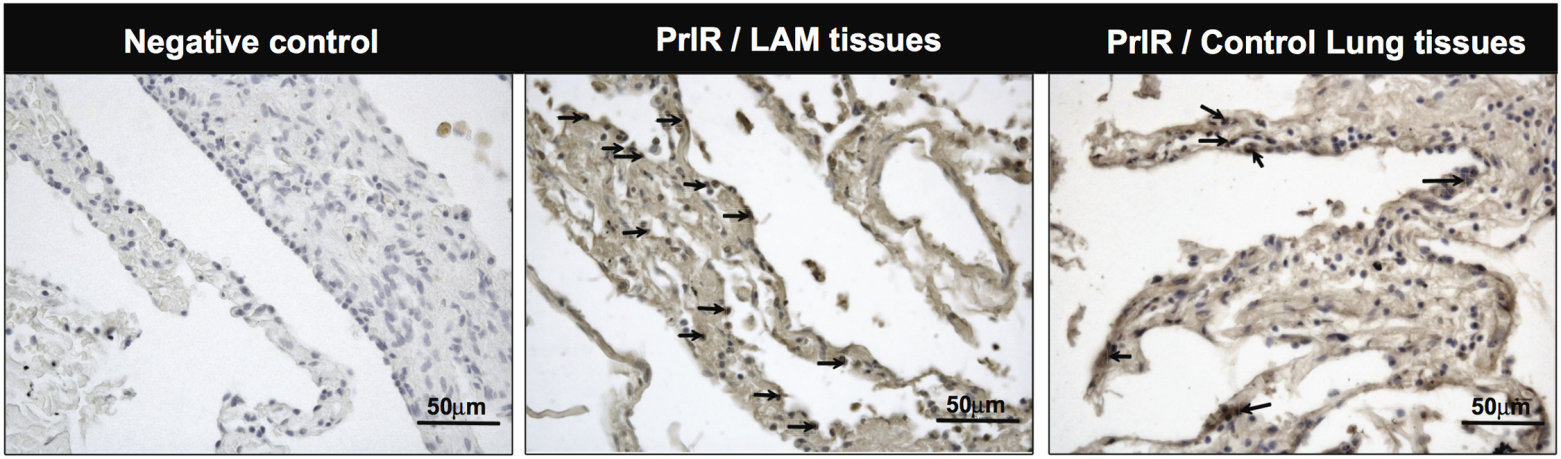
Prolactin receptor immuno reactivity in human LAM lung lesions. Histological sections from LAM patients were analyzed. Human lung tissues were obtained from subjects who underwent lung resection for diagnostic reasons and immunohistochemical-staining using the PrlR antibody clone 1A2B1 (Invitrogen) was carried out. Arrows indicate the location of PrlR immuno reactivity in one microscope field of a representative image from LAM tissue; compared to positive cells for PrlR in control lung tissue. For negative control, primary antibody was omitted, Scale bars: 50 μm.

**Fig 5:**
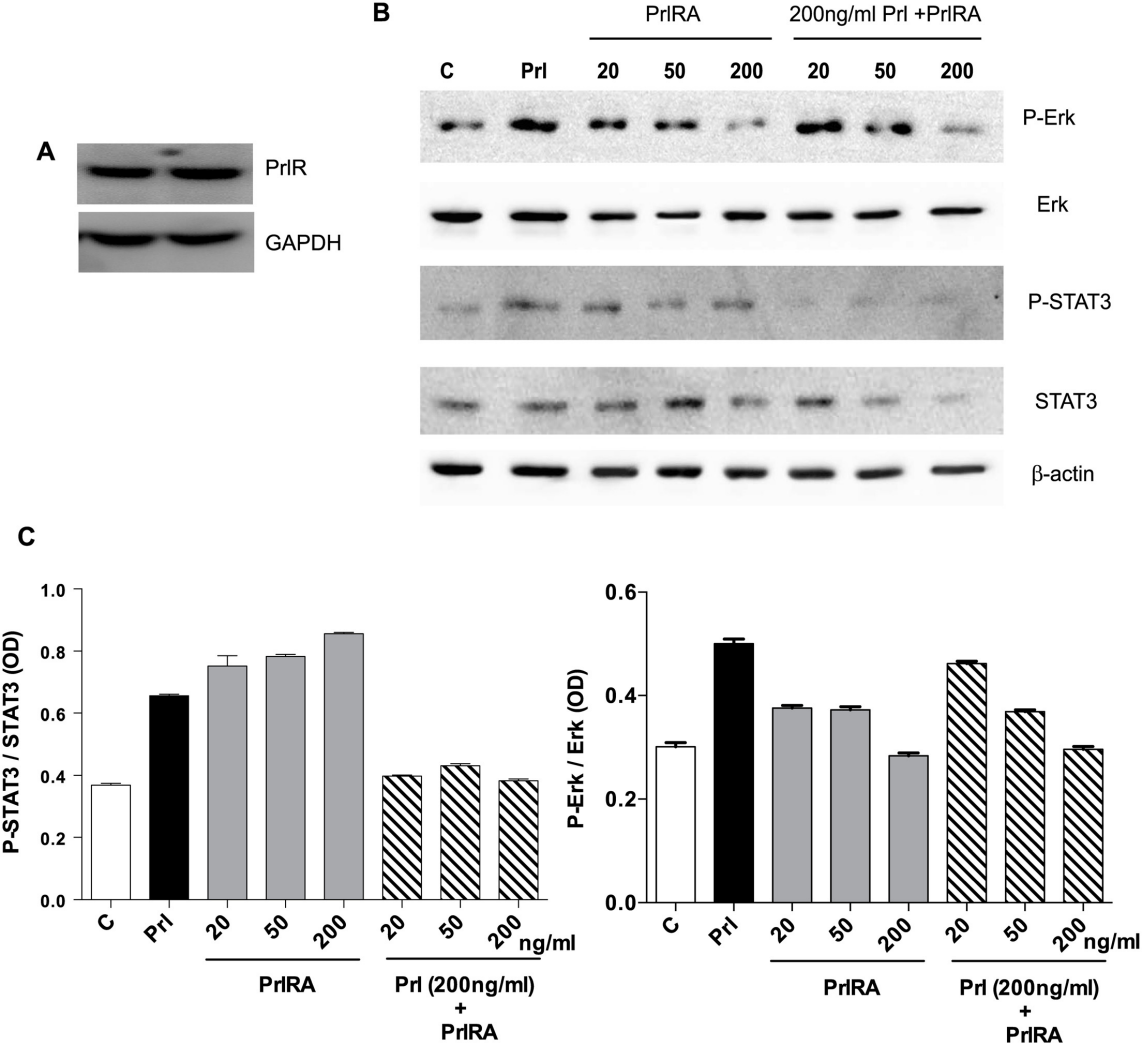
Human LAM cells express PrlR, and prolactin stimulates phosphorylation of STAT3 and Erk. LAM/TSC cells were cultured in serum-free medium overnight. The cells were then exposed to different doses of PrlRA (20, 50, 200 ng/ml) for 15 minutes, and subsequently the cells were exposed to 200 ng/ml Prl for 60 minutes or PBS as a control. Then protein extracts were prepared for Western blot analysis, and probed with antibodies for P-STAT3, STAT3, P-Erk and Erk. (A) Western blot to analyze PrlR in LAM/TSC control cells; an antibody against human PrlR detected a protein band of 89–90 kD. (B) Western blot, using antibodies directed against P-STAT3, STAT3, P-Erk and Erk in LAM/TSC cells following treatment as described in Material and methods section. (C) Densitometric quantification of Western blot signals, in which the Y-axis depicts the ratio between phosphorylated STAT3 and Erk to the total protein.

PrlR was highly expressed in the lungs of patients affected by LAM, and positivity for PrlR antibody staining was higher in LAM tissue, compared to normal lung tissue. The localization of the labeling was diffuse, and the immuno reactivity of cells within LAM lesions was variable, being localized either within nuclei or in the cytoplasm (Fig 4). Further analyses of PrlR in LAM/TSC cells using antibodies against human PrlR detected a protein band of 89–90 kD (Fig 5A).

### Prl phosphorylates STAT3/Erk, but not STAT5, in human LAM/TSC cells

The short-term effects of Prl on the phosphorylation of intracellular protein targets were tested in cells exposed to Prl. We were unable to detect any change in STAT5 phosphorylation in these cells (data not shown), whereas STAT3 and Erk were phosphorylated by Prl (Fig 5B). To block the effect of prolactin, LAM/TSC cells were starved overnight, and then incubated for 15 minutes with PrlRA (20, 50, 200 ng/ml). Prl (200 ng/ml) was subsequently added to the medium for 1 hour, after which phosphorylation of STAT3 and Erk was analysed by Western blotting (Fig 5B). In tests of the different doses of PrlRA, we found that 200 ng/ml of PrlRA decreased Prl-induced phosphorylation of STAT3 and Erk. (Fig 5B) further shows that PrlRA reduced phosphorylation of STAT3/Erk even in the absence of Prl.

### PrlR signalling stimulates LAM/TSC cell growth in a manner which can be blocked by a PrlRA

**Fig 6:**
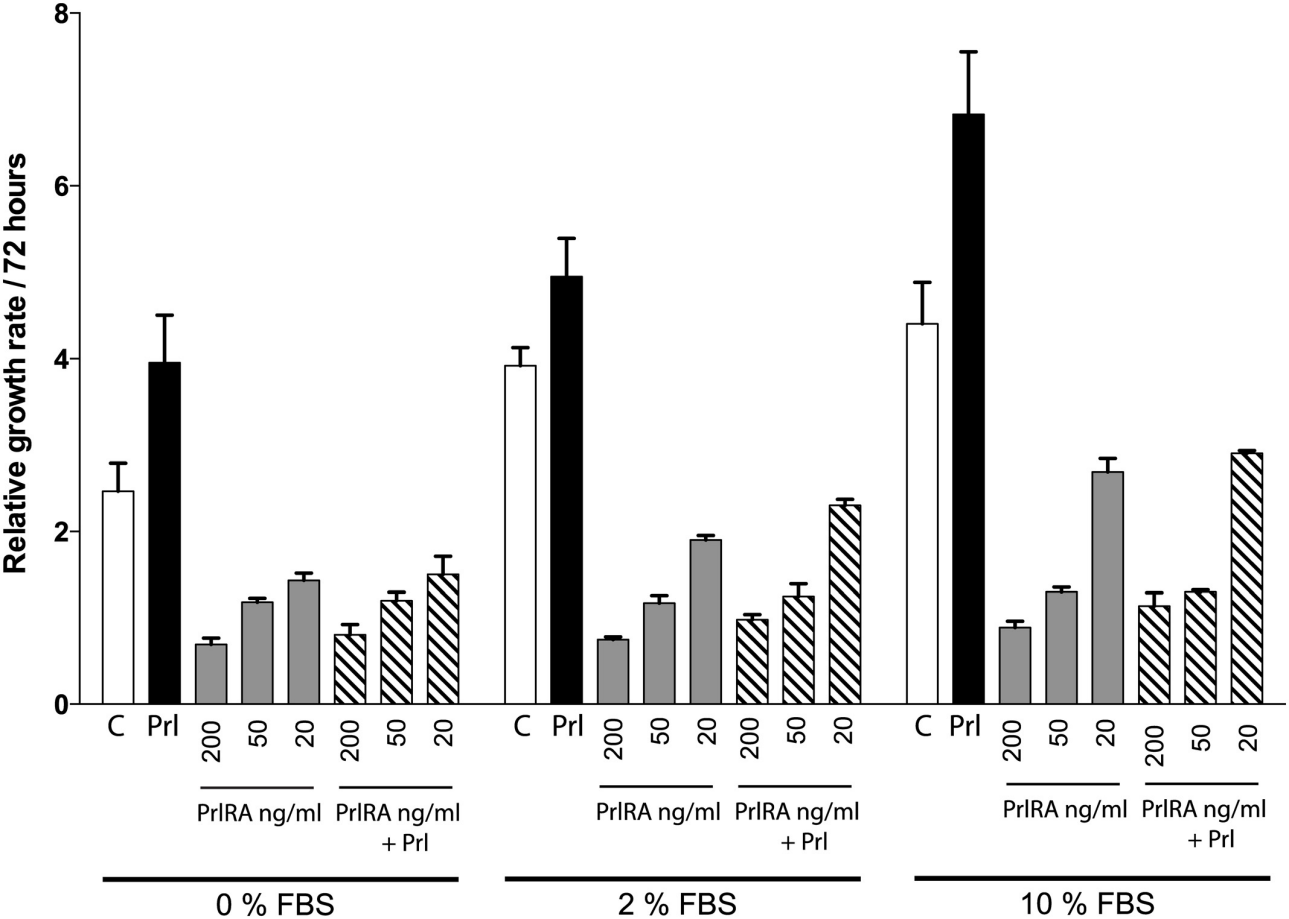
Addition of exogenous Prl stimulates cell proliferation in a manner which can be blocked by PrlRA. LAM/TSC cells were cultured in different concentrations of serum (0%, 2%, 10%). Human Prl was added to sub-confluent cells at a concentration of 200 ng/ml, with or without PrlRA (20, 50, 200 ng/ml). After 72 hours, cells were subject to the crystal violet assay. The X-axis depicts serum concentration and the Y-axis shows relative cell proliferation, as determined by absorbance at 600 nm. Each data point represents a triplicate assay. All values are +/- standard deviation, P-value <0.05.

To evaluate whether LAM/TSC cells react to Prl stimulation, cells were cultured at different serum concentrations. Addition of human Prl resulted in growth stimulation, as assessed using the crystal violet method. A response to Prl was seen at all of the serum concentrations tested, and tended to be largest in high (10%) serum concentrations (Fig 6). Addition of PrlRA (20, 50, 200 ng/ml) significantly inhibited Prl-induced LAM cell growth in a dose-dependent manner (Fig 6).

**Fig 7:**
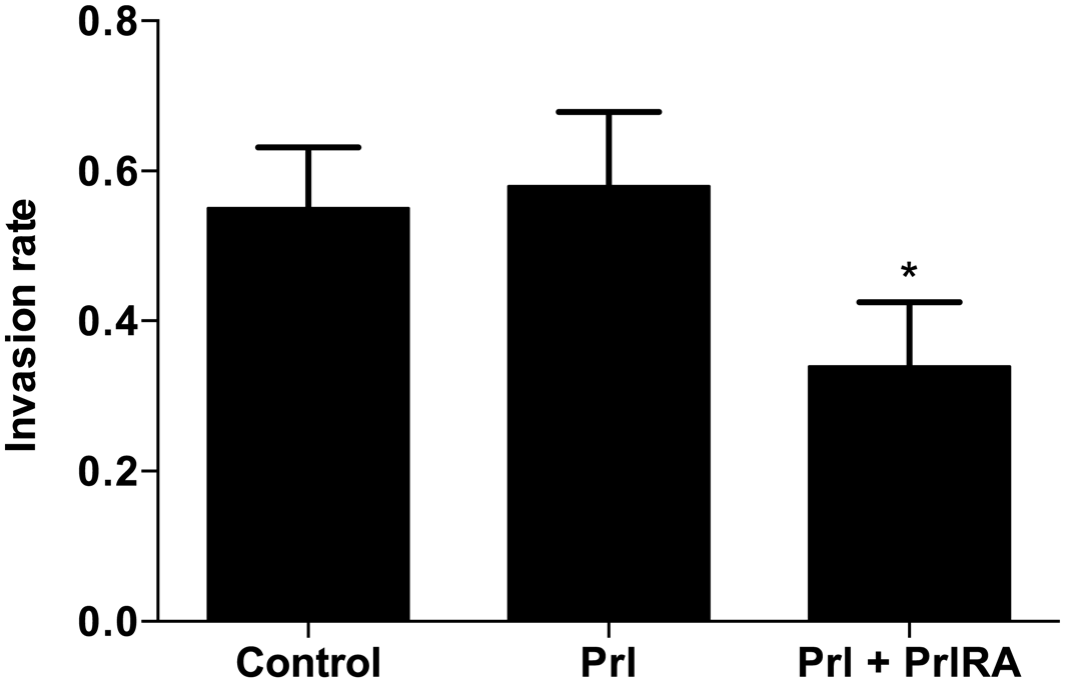
Effect of Prl and PrlRA on cell invasion. LAM/TSC cells were cultured in serum-free medium in 24-well cytoselect transwell plates. The cells were cultured with Prl or Prl and PrlRA (both at 200 ng/ml). After 48 hours, cell extracts were prepared from the layer representing invading cells, and the optical densities of the extracts were measured at 560 nm. At this dose level, Prl did not stimulate cell invasion, but the combination of Prl and PrlRA reduced invasion by 60%; * = P-value <0.05.

We studied the invasive properties of human LAM/TSC tumor cells exposed to Prl and the PrlRA. At the conditions used, Prl did not significantly stimulate cell invasion, but the combination of Prl and PrlRA decreased the extent of invasion by approximately 60% (Fig 7).

## Discussion

In this study, we show that PrlR levels are increased in a mouse sarcoma cell line treated with TSC2 siRNA. Interestingly, the GH receptor was only marginally affected by TSC2 knock-down, suggesting that changes in PrlR levels due to reductions in TSC2 expression had relatively specific effects in the cell system used. We also found that the Prl receptor is readily detectable in cells derived from human LAM lesions. These cells respond to Prl by increasing proliferation and by phosphorylation of STAT3 and Erk. The invasive capacity of human LAM cells can be reduced by use of PrlRA. Analyses of patient material from LAM patients showed that LAM lesions express PrlR. Taken together; our results support the idea that LAM cells are Prl-dependent.

A mechanistic explanation for increased Prl sensitivity in LAM cells requires further studies, but it is interesting that elimination of one signalling component (i.e. TSC2) can influence the composition of membrane proteins. For example, previous work has demonstrated that expression of integrin can be altered by changes in TSC2 [29]. TSC2 is a GTPase-activating protein which, via the targeting of RHEB, suppresses the activity of mTORC1 [8]. Consequently, elimination of TSC2 activates mTORC1 and its downstream effector signals, including S6 kinase, leading to diverse cellular responses that include increased protein synthesis. One regulator of TSC2 is AKT, and stimulation of this kinase by e.g. growth-promoting agents, destabilizes TSC2, resulting in increased mTORC1 activity [30]. Another system that regulates mTORC1 is the “energy sensor” AMP kinase that senses changes in the cellular ATP/AMP ratio [31]. Prl is known to activate AKT, and as a growth-stimulating factor, it also changes AMP kinase activity. The most well studied effects of PrlR signalling involve the JAK2 and STAT (notably STAT5) pathway, and JAK2 activation is in turn dependent upon PrlR dimerization due to Prl binding to two PrlRs. The regulation of STAT3 by Prl observed in this study confirms previous studies, and activation of this transcription factor seems to be of particular relevance for LAM cell growth; indeed, a study by Goncharova *et al*. [32] showed that phosphorylation of STAT3 is critical for the enhanced growth of LAM cells.

As previously described [32], increased STAT3 phosphorylation is also seen in TSC2-null xenografted tumors. In some cell systems, SOCS3 is an established negative regulator of STAT proteins, and elevation of SOCS3 can down-regulate membrane PrlRs [33]. In our experiments, however, we found that SOCS3 was not changed by suppression of TSC2 levels, and therefore other explanations which link reductions in TCS2 to increased Prl receptor expression are needed.

Although our experiments only concerned experimental cell models, it is interesting to speculate whether increased Prl sensitivity is of relevance for the growth of LAM lesions. A previous study by Terasaki *et al*. showed that LAM lesions produce Prl, and that patients with LAM have elevated serum Prl levels [34]. Extra-pituitary Prl production in humans, due to the presence of a separate gene promoter, has been elegantly shown by Horseman *et al*. by replacing the mouse Prl promoter with its human counterpart [35,36].Taken together, there is room to hypothesize that LAM lesions are hypersensitive to endocrine or locally produced Prl, and that LAM lesions will respond to agents disrupting Prl signals. One way to block Prl signalling is to block the Prl receptor. Prl receptor antagonists have been created by the introduction of specific amino acid changes into Prl, which prevent receptor dimerization and activation. The antagonist used in the present study is known to block Prl-induced STAT5 activation, and we observed that blocking PrlR reduced Prl-induced activation of STAT3 and Erk in LAM cells. In the present study, we observed that the PrlR antagonist could exert its action alone, i.e. in the absence of Prl. We do not have a clear explanation for this effect, but further studies concerning e.g. the local production of Prl might provide an explanation for this phenomenon. Future studies are needed to clarify the potential role of PrlR antagonists in the treatment of LAM.

In conclusion, reductions in levels of TSC2 in cells leads to increases in PrlR levels, and antagonism of this receptor may offer a new therapeutic approach in the treatment of LAM.
